# Impact of virtual embodiment and exercises on functional ability and range of motion in orthopedic rehabilitation

**DOI:** 10.1038/s41598-022-08917-3

**Published:** 2022-03-23

**Authors:** Marta Matamala-Gomez, Mel Slater, Maria V. Sanchez-Vives

**Affiliations:** 1 Institut d’Investigacions Biomèdiques August Pi I Sunyer (IDIBAPS), Rosselló 149-153, 08036 Barcelona, Spain; 2 Event-Lab, Department of Clinical Psychology and Psychobiology, Universitat de Barcelona, Passeig de la Vall d’Hebron 171, 08035 Barcelona, Spain; 3 Institute of Neurosciences of the University of Barcelona, Barcelona, Spain; 4 ICREA, Passeig Lluís Companys 23, 08010 Barcelona, Spain; 5 Present Address: Mind and Behavior Technological Center, Department of Psychology, University of Milano-Bicocca, 20126 Milan, Italy

**Keywords:** Neuroscience, Fracture repair, Orthopaedics

## Abstract

Recent evidence supports the use of immersive virtual reality (immersive VR) as a means of applying visual feedback techniques in neurorehabilitation. In this study, we investigated the benefits of an embodiment-based immersive VR training program for orthopedic upper limb rehabilitation, with the aim of improving the motor functional ability of the arm and accelerating the rehabilitation process in patients with a conservatively managed distal radius fracture. We designed a rehabilitation program based on developing ownership over a virtual arm and then exercising it in immersive VR. We carried out a between 3-group controlled trial with 54 patients (mean age = 61.80 ± 14.18): 20 patients were assigned to the experimental training group (immersive VR), 20 to the conventional digit mobilization (CDM) training control group, and 14 to a non-immersive (non-immersive VR) training control group. We found that functional recovery of the arm in the immersive VR group was correlated with the ownership and agency scores over the virtual arm. We also found larger range of joint movements and lower disability of the fractured arm compared with patients in the Non-immersive VR and CDM groups. Feeling embodied in a virtual body can be used as a rehabilitation tool to speed up and improve motor functional recovery of a fractured arm after the immobilization period.

## Introduction

Distal radius fractures are one of the most common fractures of the upper limb and occur with a ratio of about 3:1 in females compared to males^1^. After a distal radius fracture, patients are treated surgically or by casting, being immobilized for between 2 and 6 weeks^2^. The rehabilitation process after a distal radius fracture is quite complicated and most patients continue with functional deficits for up to six months post-fracture. Moreover, during the period of immobilization, patients often keep their fractured hand in rigid postures, leading to joint stiffness, nerve, tendon, and ligament problems, reduced range of motion (ROM), muscular atrophy, and loss of internal movement representation^3^. Thus, the optimal time for initiating the rehabilitation process after a distal radius fracture is during the immobilization period^4^.

Whereas conventional treatment during the immobilization period consists in early digit mobilization of the affected hand^5^, there is increasing interest in the integration of mental practice techniques in rehabilitation programs, which involve mentally rehearsing motor tasks (e.g., walking) when physical practice is not possible. Several studies have shown a shrinkage of the cortical representation of the affected limb in the primary somatosensory cortex in conditions such as some types of chronic pain^6^, immobilization of the upper limb (e.g. cast)^7^, or after an upper limb paresis because of a brain injury. The prevention of these changes is a potential therapeutic target. Moseley^8^, reported that by using mental training techniques it is possible to reduce chronic pain, allegedly by activating the cortical areas related to the affected limb^9,10^, which can lead to preservation of the cortical representation of the affected limb in the brain, and subsequent symptomatic and functional improvements^11,12^. In this regard, mental tasks such as the use of motor imagery and action observation have been recently proposed as potentially useful adjunct treatments to conventional motor learning training^13,14^.

One way of integrating motor imagery, action planning, and action observation is by using immersive virtual reality (VR), through which one can induce the illusion of owning a virtual body—or a virtual arm—when it is co-located with the real body—or real arm^15^. As a result, immersive VR is a promising tool that can allow the user to simultaneously imagine and observe motor actions of a virtual limb that is perceived as one’s own, and even allow the incorporation of devices (e.g., a pedal or a switch) that enable agency over the movement of the virtual limb. This may train and strengthen the neural network involved in motor coordination and execution and may consequently accelerate the rehabilitation process. The integration of technology at this frontier is of interest from both a medical and social perspective^16–18^, with evidence justifying the use of immersive VR in clinical applications^17,19–21^. In the field of immersive VR, it has been demonstrated that synchronous visuo-tactile correlations induce the illusion of owning a virtual arm^22^, and more generally that appropriate multisensory and/or sensorimotor correlations received on a virtual body that is seen from a first person perspective and co-located with the real body, induces an illusion of ownership over that body^23,24^. Moreover, through immersive VR we can also induce the sense of agency over the virtual arm^25^. The sense of agency is described as the attribution to the self of the control over our own movements and gives us a sense of control and responsibility over our own actions^26,27^.

In this study we investigated the impact of an immersive VR training program developed by our group and based on our previous work on embodiment of a virtual body^22–25^ and on the potential of virtual embodiment for neurorehabilitation^13^. Immersive VR allows the patient to train the illusory “owned” virtual body when the physical body is immobilised, opening up the possibility of investigating this approach for upper limb rehabilitation during a period of immobilisation.

## Materials and methods

### Participants

A between three-group study of patients with a distal radius fracture was carried out at the rehabilitation department of the Hospital Clinic of Barcelona, Spain. We recruited patients with distal radius fracture aged 18–80 years.

Diagnosis of the distal radius fracture was confirmed by x-ray and patients were recruited from the traumatology department of the hospital. Patients were excluded: if they had cognitive impairment detected by the Mini-Mental State Examination test (MMSE < 24/30) or Frontal Assessment Battery test (FAB < 12/18) to ensure that they could understand the task instructions during the training period; if they had a history of seizures or epilepsy (except for febrile seizures of childhood); or if they had another condition that put the patient at risk (e.g., visual impairments or infection). The study was approved by the local ethics committee (Comité Ético de Investigación Clínica de la Corporación Sanitaria Hospital Clínic de Barcelona), and carried out according to the Declaration of Helsinki. Written informed consent was obtained from all patients, and patients were advised they could leave the study at any time.

**Figure 1:**
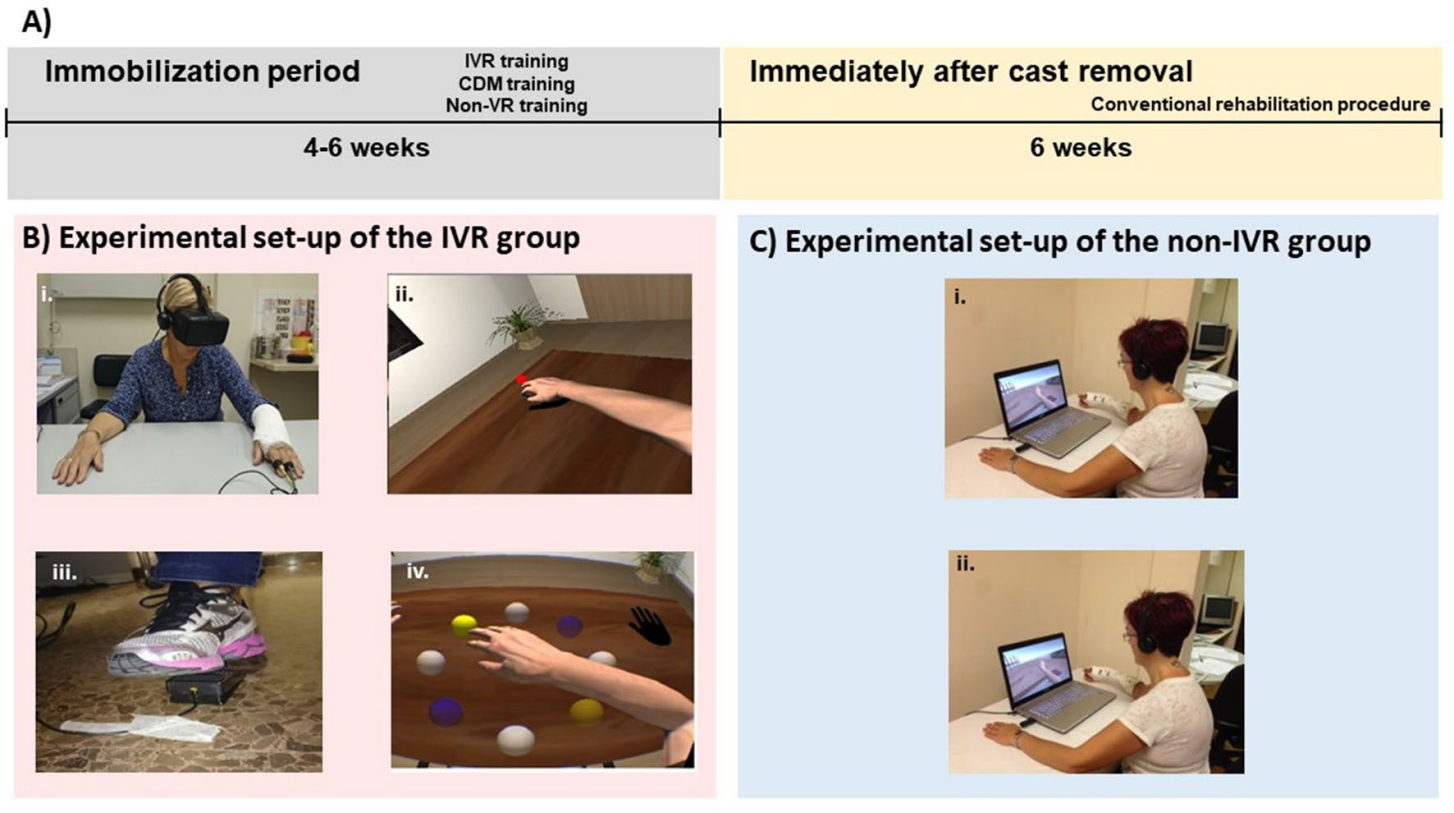
Experimental design. (**A**) Timeline of the study design. (**B**) Experimental set-up of the immersirve virtual reality (IVR) group. (i) The patient used a head-mounted display (HMD) that provides an immersive virtual environment and allows the participant to feel embodied in a virtual body viewed from a first-person perspective. They also wore headphones through which they could listen to the task instructions and movement descriptions during the whole session. The virtual arms were co-located with the real arms. (ii) The red ball repeatedly tapped the fingers during the visuo-tactile stimulation phase at the beginning of each session. Participants were asked to look at the fractured virtual arm throughout the session. Next, the patient listened to the description of the movements and then had to mentally plan the movement. (iii) Once the patient had mentally planned the movement, they had to push the pedal to activate the virtual arm movement. (iv) Finally the patient saw the virtual arm movement (visual feedback of the planned movement), from a first-person perspective. (Motor imagery/action planning + Action observation paradigm). (**C**) Experimental setup of the non-immersive VR group. (i) The patient sat in front of the computer with their arms placed above the table co-located with the virtual arms. The patient wore headphones in order to be isolated from the external noise. (ii) The patient looked at the virtual arm movements from a third-person perspective (Action observation paradigm).

Patients were randomly assigned following the randomization list provided by the randomization software (https://www.randomizer.org/), within the first week of the distal radius fracture injury to one of three different groups that carried out different training programs during the immobilization period: (1) the experimental group (*n* = 20), where patients did an immersive VR training consisting of motor planning and action observation in an immersive virtual environment; (2) a control group (*n* = 20), where patients did CDM training at home; and (3) another control group (*n* = 14) that did a non-immersive virtual reality training consisting of action observation on a computer screen (see Fig. 1B). Patients in the immersive VR and non-immersive VR groups visualized the same exercise program but in an immersive or in a non-immersive way, respectively. Patients were not aware of the existence of the other groups, although it was not a blinded study.

### Procedures

We designed a motor-cognitive training program that combined motor imagery/action planning with action observation from a first-person perspective in immersive virtual reality, based on our previous studies on virtual embodiment^22–24^ and on the process of rehabilitation carried out in physiotherapy. Our program contained a set of exercises organized into six different modules of increasing complexity with the aim of rehabilitating global upper limb mobility. Every week the physiotherapist (researcher) changed the exercise module, starting with the first one. The intensity and duration of the interventions were the same in the three groups, consisting of a 4- to 6-week training period (depending on the evolution of the fracture of each patient, although there were no between-group differences), three days per week, for 20 min. In the immersive VR and non-immersive VR training groups, a physiotherapist administered the interventions for each patient in a one-to-one session. Patients were assessed at baseline (T0), one day after the cast immobilization; after the intervention period (T1), which was immediately after cast removal; and 6 weeks after cast removal (T2) as a follow-up. The intervention period lasted from 4 to 6 weeks depending on the evolution of the patient, between T0 and T1, in the three groups (Fig. 1A). Between T1 (immediately after cast removal) and T2 (follow-up) all patients followed the usual protocol after plaster removal, doing conventional rehabilitation therapy. Immersive VR training was performed using a head-mounted display (HMD) and vibrators on the fingers and hand of the patients to augment the feeling of ownership of the virtual body, through visuotactile correlations, a method described by Slater et al.^22^ We used a head-mounted display (HMD; Rift Development Kit 2, Oculus, Menlo Park, CA, USA) with a resolution of 960 × 1080 pixels per eye and a nominal field of view of 100°, displayed at 75 Hz to show the virtual environment, which was programmed in Unity 4.5.3 (Unity Technologies, San Francisco). The virtual male and female body were taken from the Rocketbox library (Rocketbox Studios GmbH, Hannover). The HMD was connected to a laptop. In the non-immersive VR training group, patients visualized the same program used in the immersive VR training on the laptop screen without using the HMD (i.e., in a non-immersive way). As said above, we used three vibrators in order to induce embodiment (i.e., the illusion of ownership) over the virtual body through visuo-tactile correlations in the immersive VR group: two vibrators were attached to the dorsal distal phalanges of the right index and middle fingers and the third vibrator was attached to the palm of the hand of the patients. The vibrators were controlled by the same Unity program that controlled the visual input through an Arduino controller. Vibrations had a duration of 1 s. There was no visuo-tactile stimulation in the non-immersive VR training group. Headphones were used in order to allow the patients to follow the task instructions of each exercise in the immersive VR training group. In the non-immersive VR training group, headphones playing pink noise were used to isolate the patients from the environmental noise. Patients in the immersive VR training group were first familiarized with the virtual environment from a first-person perspective through the HMD. Patients were instructed to look around the virtual room, to describe what they saw, and to look down at the virtual body sitting on a chair that was seen from the first-person perspective to be visually substituting the patient’s own body. Patients in the immersive VR and the non-immersive VR groups sat comfortably on a chair with both arms resting on a table in front of them. They were instructed not to move their arms during the training sessions. Patients in the immersive VR training group saw a virtual male or female body (corresponding to the sex of the patient) co-located with their own body, seen from a first-person perspective through the HMD and with their virtual arms resting on a table in front of them in the same position as the real arms. Participants were then asked to concentrate on their injured virtual hand. They saw a ball tapping in random order the virtual index and middle fingers of the injured hand and felt synchronous tactile feedback (vibration) on their real index and middle fingers of the injured. In addition, we placed a pedal under the foot of patients in the VR group that was used to initiate the virtual arm movement in each exercise; this allowed the patients to choose when they were ready to perform the exercise, inducing agency over the virtual arm movements. The immersive VR task involved listening to task instructions describing the movement to be done through the headphones so that the patient could mentally plan each exercise. After this, they had to push the pedal placed under their foot to activate the virtual arm. Finally, patients observed their virtual arm doing the exercises from a first-person perspective (Fig. 1Bv). In some exercises, patients had to interact with virtual balls or different virtual objects as targets of the task; when they reached the object, they felt a vibration on the palm of their injured hand to enhance sensory feedback. Of course, they were not required to move their real arm, but the virtual arm moved for them. Patients in the CDM training group only followed the doctor’s directions, which consisted in opening and closing their hand 20–30 times per day and mobilizing their fingers at home. Patients in the non-immersive VR group saw a virtual male or female arm (corresponding to the gender of the patient) represented as being in the same position as their own arms in a computer screen. The patients were first familiarized with the virtual environment, they were instructed to concentrate only on the movements they saw on the screen placed in front of them. We did not use a pedal in the non-immersive VR training group as they only did action observation training. Thus, in the non-immersive VR training group, they observed the virtual arm doing the exercises on the computer screen, in a non-immersive virtual reality scenario (Fig. 1C).

After each session, the immersive VR training group and the non-immersive VR training group completed a six-item experience questionnaire in Spanish. Each question was scored according to a five-point Likert Scale, 1 meaning ‘totally disagree’ and 5 ‘totally agree’ (see Supplementary Material, VR questionnaire).

### Assessed rehabilitation outcomes

The primary outcome measure was the recovery of the functional ability of the arm after cast removal as measured using the Fugl-Meyer (FM) test^28^. We only assessed the upper limb section. The FM test enables a volitional movement assessment (see “Results”).

Secondary outcomes after cast removal were: percentage of disability of the fractured arm assessed using the Disability of the Arm, Shoulder and Hand (DASH) questionnaire^29^, which is a 30-item, self-reported questionnaire designed to measure physical function and symptoms in patients with musculoskeletal disorders of the upper limbs when performing functional activities and daily-life tasks; range of motion (ROM) improvement in six different movements: wrist flexion/extension, wrist ulnar and radial deviation, and pronation/supination of the forearm, all measured with a goniometer^30^.

### Statistical analysis

We first explored whether the data were normally distributed with the Kolmogorov–Smirnov test (*p* > 0.05). We used the Kruskal–Wallis test if the data were not normally distributed and one-way ANOVA (one factor: “group” with three groups) if the data were normally distributed. In order to identify possible changes associated with the implementation of the IVR training, the analysis of data was carried out using one-way ANOVA (one factor, “group”). The analysis was done separately at times T1 and T2 to study the outcome differences between IVR, CDM and Non-IVR training groups. As patients in the three groups did conventional rehabilitation training between T1 and T2, we expected to see a natural motor functional improvement in the fractured arm; for this reason we felt it was unnecessary to do repeated-measures analysis. In other words, the main focus of our study was to see the differences between groups at each specific assessment time (T1 and T2). Post-hoc analyses were conducted with the Tukey HSD test. The significance level was set at *p* < 0.05. We compared the scores reported for the “virtual reality experience” questionnaire between groups with the Mann–Whitney U test. We explored the relationship between the recovery of the functional ability of the fractured arm (FM test) and the scores obtained in the virtual reality experience questionnaire with Spearman’s correlation test. Statistical comparisons between groups were conducted with Stata version 13 (StataCorp LP, College Station, TX, USA; https://www.stata.com/).

## Results

Baseline balance between groups was confirmed for all demographics except for gender as there were more females than males in the study (Table 1), in line with distal radius fracture being more common in females^1^.

**Table 1:** Baseline characteristics of the intention-to-treat population (summarizes mean, SD and, percentages (%)).

	Immersive VR	CDM	Non-immersive VR
**Sex**			
Male	0 (0%)	3 (15%)	5 (35.71%)
Female	20 (100%)	17 (85%)	9 (64.29%)
Age (years)	60.05 ± 12.84	61.60 ± 16.23	64.57 ± 13.46
MMSE	34.70 ± 1.03	34.02 ± 1.50	34.35 ± 0.84
FAB	17.20 ± 0.90	16.50 ± 1.20	17.14 ± 1.23
Training weeks	3.20 ± 2.32	Training at home	2.64 ± 2.50

*MMSE* mini mental state examination, *FAB* frontal assessment battery.

### Motor functional ability and percentage of arm recovery

**Figure 2:**
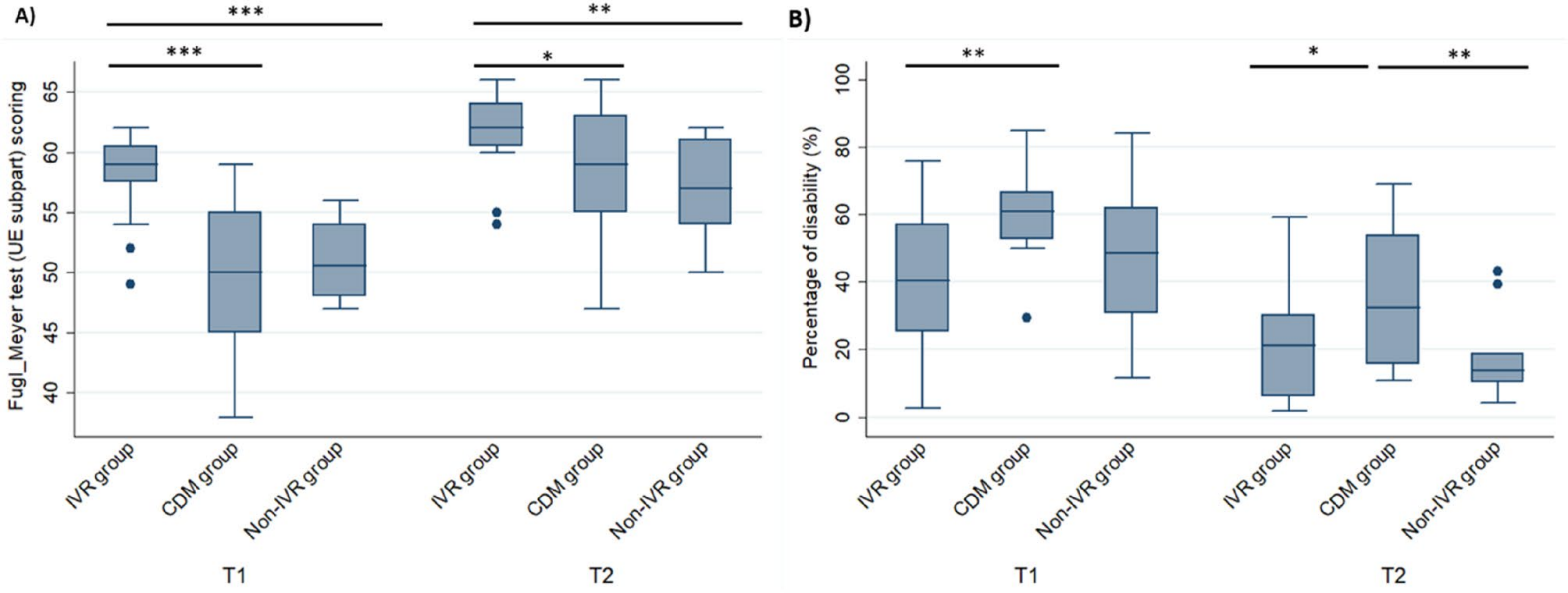
Functional ability recovery and decrease in disability scores. (**A**) Functional ability recovery. The immersive VR training group presented higher functional motor ability recovery after cast removal (T1) and six weeks later (T2) than CDM and non-immersive VR training groups, assessed by Fugl-Meyer test. (**B**) Upper limb disability scores. IVR group presented lower arm disability scores after the cast removal (T1) than CDM and Non-IVR groups, assessed using the DASH questionnaire. At T2 immersive VR and non-immersive VR groups presented lower arm disability scores compared with the CDM training group. In the boxplots, the medians are shown as horizontal lines and the boxes are the interquartile ranges (IQR). The whiskers are between max (min score, lower quartile -1.5 IQR) to min (max score, upper quartile + 1.5 IQR). If there are values outside the whiskers these are conventionally called “outliers” and are shown by (∘).

The primary outcome measure was the recovery of the functional arm’s ability after cast removal measured using the Fugl-Meyer (FM) test^28^. The FM tests volitional movement assessment including flexor synergy, extensor synergy, movement combining synergies, movement out of synergy, wrist mobility, hand grip strength, and arm coordination/speed. Thus, we assessed the prognostic of the functional ability recovery using the FM test, where a score ≤ 33 indicates a poor prognostic recovery, 34-56 a moderate prognostic recovery, and ≥ 57 a good prognostic recovery of the functional ability^28^. A higher percentage of patients in the immersive VR training group presented better prognostic recovery of the functional ability of the fractured arm after cast removal and six weeks later compared with patients in the conventional digital mobilization (CDM) and in the non-immersive VR groups (Fig. 2A) (one-way ANOVA, factor “group”: *F* = 20.83, *p* < 0.0001). More specifically, 85% of patients in the immersive VR group presented good prognostic recovery (score ≥ 57) and only 15% presented moderate prognostic recovery (score < 57) of the functional ability after cast removal (T1; 95% CI 56.84–59.96). Only 25% of patients in the CDM training group presented a good prognostic recovery of the functional ability after cast removal and 75% presented a moderate prognostic recovery (95% CI 46.52–52.48). Finally, 100% of the patients allocated in the non-immersive VR training showed a moderate prognostic recovery of the functional ability of the fractured arm at T1 (95% CI 48.95–52.47). Overall, patients in the immersive VR training group obtained higher scores in FM test compared to those in the CDM (Tukey post-hoc test: *p* < 0.0001) and Non-immersive VR (*p* < 0.0001) groups. Six weeks later (T2), patients in the immersive VR group still had better prognostic recovery (one-way ANOVA, factor “group”: *F* = 5,873, *p* = 0.005). In particular, 90% of patients in the immersive VR training group again showed good prognostic recovery of the functional ability while 10% presented moderate prognostic recovery (95% CI 60.42–63.28). In contrast, 60% of the patients in the CDM training group presented good prognostic recovery and 35% continued to show moderate prognostic recovery of the functional ability of the fractured arm (95% CI 55.69–60.94). In the Non-immersive VR training group, 50% of the patients presented good prognostic recovery and the other 50% presented moderate prognostic recovery of the functional ability of the fractured arm (95% CI 54.38–59.47). Again, patients in the immersive VR training group had better functional recovery compared with CDM (Tukey: *p* = 0.038) and non-immersive VR (*p* = 0.007) groups.

Using the Disability of the Arm, Shoulder and Hand (DASH) questionnaire^29^, we measured physical function and symptoms in the patients when performing functional activities and daily-life tasks. Patients in the immersive VR training group presented a significantly lower arm disability score in performing daily life activities at home after cast removal (T1; one-way ANOVA factor “group”: *F* = 6.224, *p* = 0.004) and a substantial decrease in the percentage of disability compared to CDM training at T2 (Fig. 2B) (one-way ANOVA at T2 factor “group”: *F* = 5.835, *p* = 0.005). More specifically, the immersive VR group presented a lower percentage of disability of the fractured arm after cast removal compared to the CDM group at T1 (Tukey: *p* = 0.003) and at T2 (*p* = 0.025); additionaly, the non-immersive VR group showed better results than the CDM training group at T2 (*p* = 0.009), with less disability in patients in the immersive VR and non-immersive VR training groups (Fig. 2B).

### Range of motion improvements

**Figure 3:**
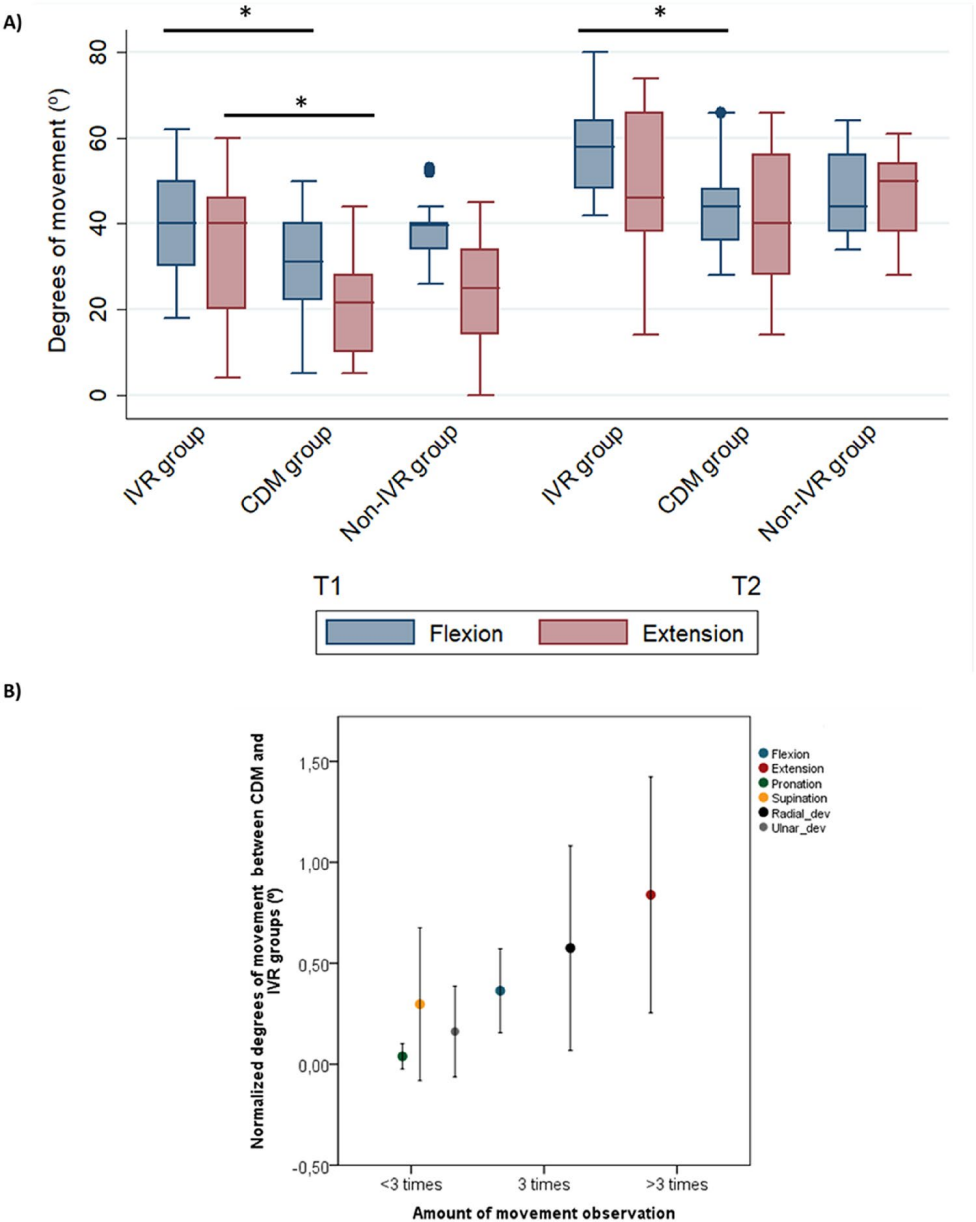
Range of motion improvement and the relationship with the amount of movement visualization. (**A**) In the range of motion assessment, the immersive VR training group presented more degrees of movement in wrist flexion/extension movements after cast removal (T1) compared to the CDM group. Again, six weeks later (T2) the immersive VR group presented greater degrees of motion than the CDM group in wrist flexion movement. The lines show the difference of degrees of motion for each different movement between groups in the T1 and T2 assessments. Error bars represent 95%CI. *p < 0.05, **p < 0.01. (**B**) The amount of movement visualization is linked with range of motion improvement. Differences between the immersive VR and CDM groups are possibly due to patients in the immersive VR group visualizing joint movements during the training period; furthermore, the patients in immersive VR group presented higher recovery in those movements that they visualized more times during the training period. Error bars represent 95%CI of the mean.

In terms of range of motion, we observed better improvements in patients in the immersive VR training group in wrist flexion (one-way ANOVA factor “group”: *F* = 4.121, *p* = 0.023) and wrist extension (*F* = 3.926, *p* = 0.027) joint movements, with a trend towards improvement in pronation (*F* = 2.933, *p* = 0.063) after cast removal (T1) compared with those in the CDM and Non-immersive VR training groups (Fig. 3A). Post-hoc analyses revealed differences only between immersive VR and CDM training groups at T1 in wrist flexion (*p* = 0.028) and in wrist extension movements (*p* = 0.021), and a trend between immersive VR and non-immersive VR groups in pronation movement (*p* = 0.056). Likewise, at T2 we found significant differences between groups in wrist flexion (*F* = 7.63, *p* = 0.001) and radial deviation (*F* = 4.40, *p* = 0.025) movements. Post-hoc analysis confirms that patients who underwent immersive VR training presented greater improvement in wrist flexion movement than those who did CDM training (Tukey: *p* = 0.001). However, those who did non-immersive VR training presented significantly higher degrees of movement in radial deviation movement than CDM training but not higher than the immersive VR training group (Tukey: *p* = 0.023) (see Fig. 3B). Patients that had visual movement feedback showed significantly better recovery of range of motion in immersive VR training than those that did the CDM training and, moreover, since they showed greater improvement in some specific movements, we recounted the number of times each of the six joint movements appeared throughout the training program in each exercise. As we only found significant differences in range of motion improvement after cast removal (T1) between the immersive VR and the CDM training groups, we calculated the difference in range of movement improvement between the immersive VR and CDM training groups and normalized it with the degrees of movement obtained in the CDM training group at T1. The normalization equation is:

[(degrees of movement in immersive VR (T1) – degrees of movement in CDM (T1)) / degrees of movement in CDM (T1)]. A linear regression analysis (‘normalized degrees of movement’ as dependent and ‘movement visualization’ as independent variable), showed a significant positive relationship between both variables during the immersive VR training program (Fig. 3B; Beta = 0.246, *p* = 0.001).

### Positive relationship between subjective experience and arm functional ability

**Figure 4:**
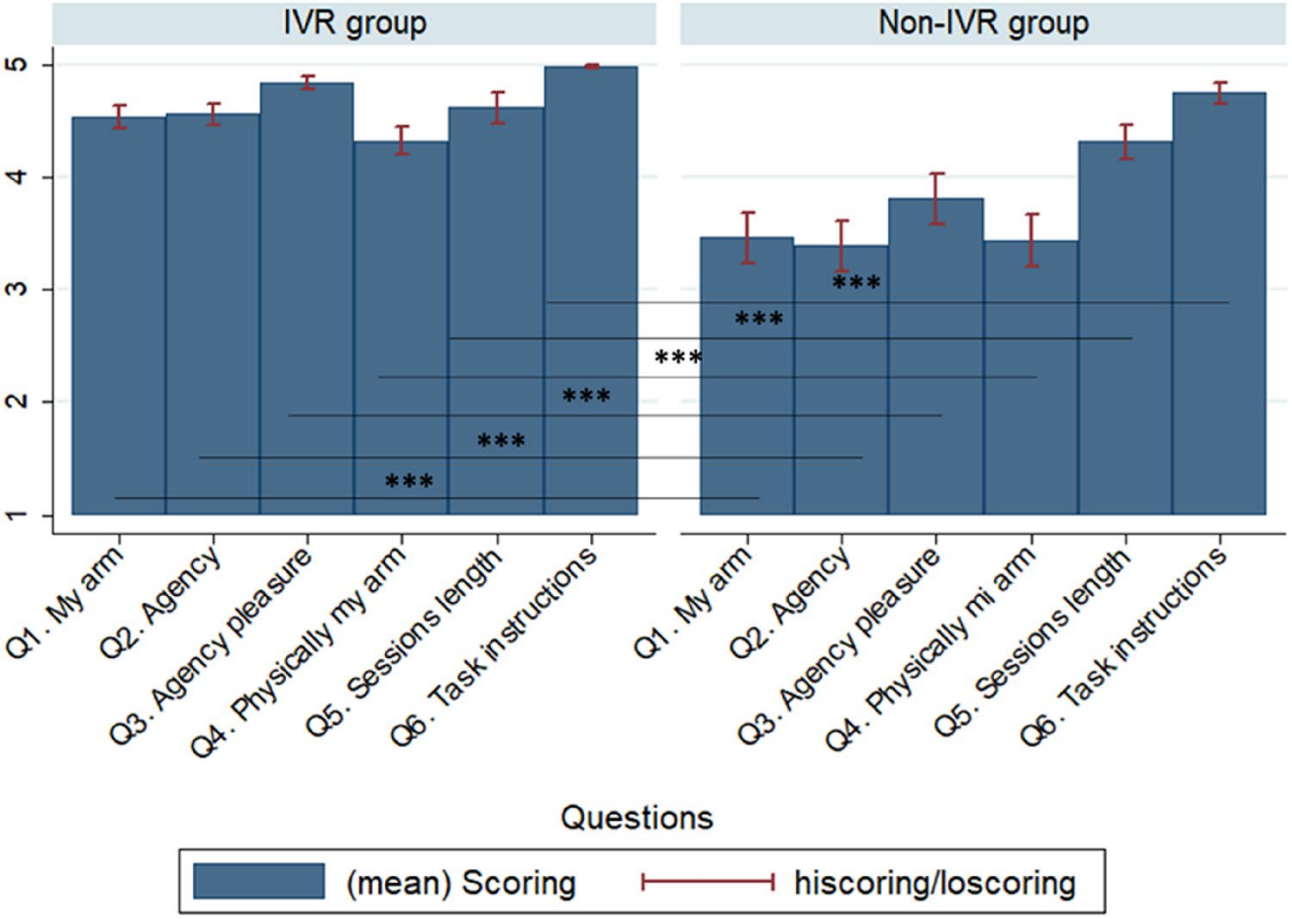
Virtual reality questionnaire differences between immersive VR and non-immersive VR groups and the relationship with functional ability recovery. Virtual reality questionnaire score differences between immersive VR and non-immersive VR groups. There were significant differences between groups for all questions of the virtual reality questionnaire, showing higher scores in questions related to the sense of ownership of the virtual body and the sense of agency by controlling the virtual arm movement. The graph also presents higher scores with respect to the appropriateness of the session duration, and clarity of the task instructions during the training sessions. Hiscoring = high scoring)/loscoring = low scoring.

Patients in the immersive VR training group reported much higher scores in the VR questionnaire that referred to their subjective experience after the training sessions (T1) compared with those in the non-immersive VR training group (Fig. 4). The most important differences were in embodiment (owning the virtual body) (one-way ANOVA, factor “group”: Q1: *p* < 0.0001; and Q4: *p* < 0.0001) and in agency (the sense of controlling the virtual arm) (Q2: *p* < 0.0001; and Q3: *p* < 0.0001). Additionally, the immersive VR group reported higher level of pleasure of the sessions’ duration and the understanding of the task instructions (Q5: *p* = 0.002; Q6: *p* < 0.0001). Interestingly, we found a positive relationship with the results obtained in the VR questionnaire and functional motor ability recovery (FM test) in the immersive VR training group at T1 (Table 2).

**Table. 2:** Relationship between the functional ability recovery of the arm after the cast removal (T1) with virtual reality questionnaire scores in immersive VR training group.

Questions	Correlation coefficients	*p*-values
Q1. My arm	0.308***	< 0.0001
Q2. Agency	0.317***	< 0.0001
Q3. Agency pleasure	0.352***	< 0.0001
Q4. Physically my arm	0.217***	< 0.0001
Q5. Sessions length	0.164**	< 0.0001
Q6. Task instructions	0.309***	< 0.0001

There was a strong positive correlation between being embodied in a virtual body and the sense of controlling the virtual arm movements with the functional recovery of the fractured arm after the cast removal in immersive VR training patients.

## Discussion

To our knowledge, this study is the first to investigate the effects of immersive virtual reality training on upper limb orthopedic rehabilitation during the immobilization period in patients with distal radius fracture, while being fully embodied in a virtual body. Our main finding is that immersive VR training, combining motor imagery through action planning and action observation, significantly improves the functional ability of the fractured arm during the immobilization period and accelerates the rehabilitation process in patients with distal radius fracture compared with non-immersive VR (active control) and CDM training. Remarkably, functional recovery was highly correlated with the feeling of ownership of, and agency (sense of control) over, the virtual arm. Consistent with these findings, patients who did the immersive VR training had lower disability scores after cast removal (T1) and six weeks later (T2) compared with CDM training; improved range of motion, especially in wrist flexion/extension movements, was also observed after cast removal compared with CDM training. Finally, we observed a non-significant trend towards lower pain ratings in patients that underwent immersive VR and non-immersive VR training compared with those that did the CDM training. The results obtained here highlight the potential of being fully embodied^22–24^ in a healthy virtual body while performing immersive VR training based on action planning and action observation for improving motor functional ability and for accelerating the rehabilitation process of patients not only with fractures but potentially also with other musculoskeletal and neurological conditions.

The finding that mental training using immersive VR from a first-person perspective attenuates physical and functional impairments in the upper limb after the immobilization period to a greater extent than non-immersive VR or conventional CDM training may be explained by different factors. First, by the repeated activation of the neural networks involved in the sensorimotor loop through action planning and action observation, which may help to prevent potential plastic changes occurring in the brain during immobilization periods, such as cortical shrinkage of the injured arm representation. Such circuit training would be more effective in situations of body ownership and control of the virtual arm, which did not occur in the non-immersive VR nor CDM groups. From the results of this study we speculate that the feeling of ownership toward a virtual body may increase sensorimotor activation when the embodied virtual body performs movements over which the subject has a sense of agency. We can also speculate that there is a descending impact on the autonomic system and muscles, as has been reported for mental imagery of movement^31,32^, enhancing heart rate, respiratory rate, and skin and muscle blood flow through cholinergic vasodilation. For example, seeing an illusory owned body exercise in immersive VR has been reported to enhance arousal, measured using skin conductance^33^.

The immersive VR rehabilitation system presented here includes the internalization (and ownership) of whole bodies. Nowadays, most current virtual rehabilitation systems for motor rehabilitation do not integrate immersive virtual environments (i.e., use non-immersive VR)^34,35^ or they present isolated virtual representations only of the tracked hands while interacting with the virtual environment^36–38^. Others have used full virtual body illusions but include a very small sample size, and with the interaction of other robotic devices^39^. In our study patients in the immersive VR group not only developed the illusion of ownership over the virtual arm, but they also controlled the initiation of the virtual arm movement in each exercise, leading to an increased sense of agency over the virtual arm, therefore allowing the patients to be more than mere spectators and instead to become active actors (mentally and physically present) within the virtual environment.

The study has some limitations. First, there was no balance between male and females among the training groups and, furthermore, there were no males in the immersive VR group; however, there is evidence that justifies a large number of females with distal radius fracture compared to males with a ratio of about 3:1. Another limitation is that we did not control the amount of digit mobilization in the CDM group; nevertheless, this is the normal procedure after distal radius fracture where patients follow the physician’s directions. As one of our interests in this study was to compare our immersive VR training with the conventional procedure, we did not measure adherence to the CDM exercise protocol.

The results of this study suggest that immersive VR can be used as an orthopedic rehabilitation tool to accelerate the motor functional recovery of the fractured arm, and more generally, of an immobilized extremity, to relearn motor skills and/or reduce atrophy in patients without movement in their upper or lower limbs due to fractures or other musculoskeletal or neurological impairments.

## Supplementary Information

Supplementary Information.
