# Supplementary material for: Impact of virtual embodiment and exercises on functional ability and range of motion in orthopedic rehabilitation
Source: Sci Rep. 2022 Mar 23;12:5046. doi: 10.1038/s41598-022-08917-3 (PMC8943096; doi:10.1038/s41598-022-08917-3)
Supplement: Supplementary file 1 — Supplementary Information. [file 41598_2022_8917_MOESM1_ESM.docx]

**Supplementary Material**

**Impact of virtual embodiment and exercises on functional ability and range of motion in orthopedic rehabilitation**

**Marta Matamala-Gomez ^a,^*, Mel Slater ^b,c^ and Maria V. Sanchez-Vives ^a,b,d^***

^a^ Institut d’Investigacions Biomèdiques August Pi i Sunyer (IDIBAPS), Rosselló 149-153, 08036 Barcelona, Spain

^b^ Event-Lab, Department of Clinical Psychology and Psychobiology, Universitat de Barcelona, Passeig de la Vall d'Hebron 171, 08035 Barcelona, Spain

^c^ Institute of Neurosciences of the University of Barcelona, Barcelona, Spain.

^d^ ICREA, Passeig Lluís Companys 23, 08010 Barcelona, Spain

***** Correspondence: Marta Matamala-Gomez, Maria V. Sanchez-Vives

***Virtual reality questionnaire***

Most of the questions of the virtual reality questionnaire were adapted and translated from Slater et al. [1] and new questions related to the understanding of the task instructions and to the duration of the sessions were added (Table S1).

**Table S1. Virtual reality questionnaire**

| **Questions** |  |
| --- | --- |
| Q1 | I felt as if the arm I saw in the virtual world was my arm. |
| Q2 | I felt I controlled the virtual arm as if it were my own arm. |
| Q3 | I liked being able to control the movements of the virtual arm. |
| Q4 | During the experiment there were moments in which the virtual arm began to resemble my own real arm in some physical aspects. |
| Q5 | The duration of the sessions seemed appropriate. |
| Q6 | The task instructions were specific and clear. |

***Flow chart of patients***

We screened 104 patients between March 1, 2014, and September 30, 2016. The most common reason for exclusion from the study was the presence of cognitive impairment (27 [25.96%] of 104 patients). Of the screened individuals, 77 patients were randomly assigned to different groups: 29 patients were assigned to the IVR training group, 27 patients to the CDM training group (control one) and 21 to the Non-IVR training group (control two). Twenty-five (86.20%) of 29 patients in the IVR group, 23 (85.18%) of 27 patients in the CDM group and 14 patients (66.66%) of 21 patients in the Non-IVR group started the training sessions; 20 (80%) of 25 patients in the IVR group, 20 (86.95%) of 23 patients in the CDM group and 14 (100%) of 14 patients in the Non-IVR group completed the training period, which lasted between four to six weeks depending on the evolution of the patient, and were included in the analysis (Fig. S1).


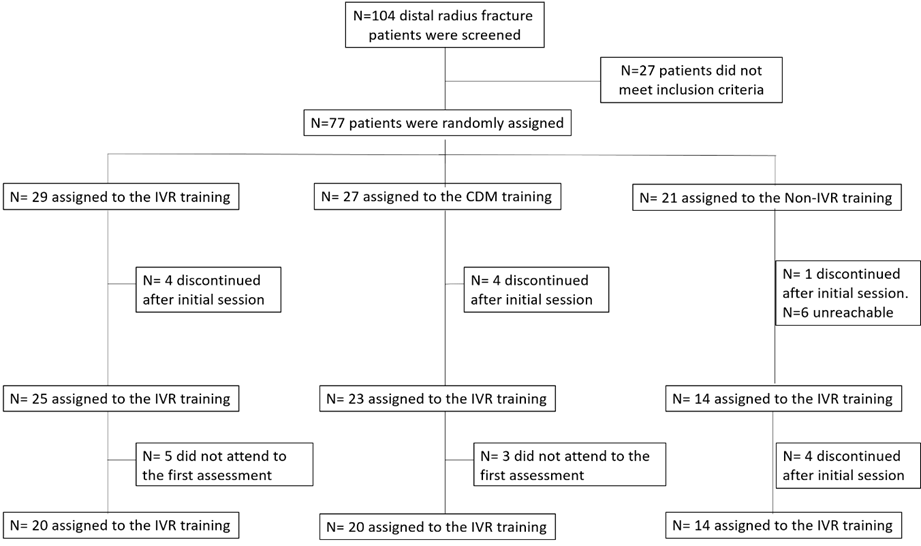


**Fig. S1. Flow chart of patients.** Seventy-seven patients were included in the intention-to-treat analysis, nine patients were discontinued after the initial session and six were unreachable. 54 subjects completed the whole study period (completed training and assessments at T1 and T2). In this paper, all of the results reported are based on this final sample size. The first study subject was recruited in March 2014. The last subjected completed the final follow-up assessment in September 2016.

[1] Slater M, Perez-Marcos D, Ehrsson HH, Sanchez-Vives M V. Towards a digital body: the virtual arm illusion. Front Hum Neurosci 2008;2:6. https://doi.org/10.3389/neuro.09.006.2008.
